# Farmers’ Preference for Rice Traits: Insights from Farm Surveys in Central Luzon, Philippines, 1966-2012

**DOI:** 10.1371/journal.pone.0136562

**Published:** 2015-08-28

**Authors:** Alice G. Laborte, Neale C. Paguirigan, Piedad F. Moya, Andrew Nelson, Adam H. Sparks, Glenn B. Gregorio

**Affiliations:** 1 Social Sciences Division, International Rice Research Institute (IRRI), Los Baños, Laguna, Philippines; 2 Crop and Environmental Sciences Division, IRRI, Los Baños, Laguna, Philippines; 3 Plant Breeding, Genetics and Biotechnology Division, IRRI, Los Baños, Laguna, Philippines; Fujian Agriculture and Forestry University, CHINA

## Abstract

Many modern rice varieties (MVs) have been released but only a few have been widely adopted by farmers. To understand farmers’ preferences, we characterized MVs released in the Philippines from 1966 to 2013 and identified important characteristics of the varieties that were widely adopted in Central Luzon using farm surveys conducted in 1966–2012. We found that farmers adopt MVs that are high yielding, mature faster, and have long and slender grains, high milling recovery, and intermediate amylose content. The amylose content of adopted varieties has been declining, suggesting value in developing softer rice. To have a high potential for adoption, new MVs should have characteristics within the ranges of values observed for the adopted MVs. In addition, new MVs should have higher head rice recovery, less chalky grains, and better resistance to pests and diseases. Most MVs released in 2005–2013 compared poorly in these three traits. To reduce the risk of severe outbreaks, broad spectrum resistance should be incorporated into new MVs. This analysis of five decades of farm surveys provides insights into the varietal characteristics preferred by farmers which could contribute to the establishment of a product profile for developing improved MVs that are more targeted and, hence, would have high potential for adoption by farmers in Central Luzon and similar areas. We recommend a similar analysis be done in other major rice growing regions to aid the development of MVs that are more responsive to farmers’ needs and preferences.

## Introduction

Aside from being the staple food, rice and its production is an important source of employment and livelihood in rural areas in the Philippines. In 2013, total rough rice production in the country was 18.4 million t from 4.7 million ha of rice land [1].

The use of modern rice varieties (MV) together with critical inputs such as fertilizer and irrigation formed the basis for the Green Revolution in Asia which brought about large increases in rice yield. The first MV, IR8, was released in 1966 and by 1980, about 40% of the total rice area in South and Southeast Asia was already planted to MVs, with a rapid adoption of the MVs observed in the Philippines than in any other country [2]. In fact, in 1966, 3% of the total rice area in the Philippines was already planted to MVs and this increased to over 75% by 1980 and to 90% by 1990 [3].

Since the mid 1960s, many rice varieties have been developed and officially released in the Philippines. Peng and Khush [4] classified varieties by decade of release and cited the emphasis of the breeding program at the International Rice Research Institute (IRRI) during each decade: dwarfism for varieties released in the 1960s, multiple disease and insect resistance in the 1970s, grain quality in the 1980s, and high yield with hybrid rice and the new plant type in the 1990s. Similarly, Launio et al. [5] adapted the classification from Estudilo and Otsuka [6] to group MVs based on their dates of release and distinct characteristics. The first-generation MVs (MV1) were released from the mid 1960s to the mid 1970s and included IR5 to IR34 developed by IRRI and the C4 series developed by the University of the Philippines. These were semi-dwarf varieties and were more responsive to fertilizers than traditional varieties (TVs). They were also potentially higher yielding than TVs but were susceptible to pests and diseases. Second-generation MVs (MV2) were released from the mid 1970s to the mid 1980s and included IR36 to IR62. MV2s have multiple pest and disease resistance, making yields more stable. The third-generation MVs (MV3) consisted of IR64 to IR72 and PSB Rc varieties that were released from the mid 1980s to the mid 1990s. These varieties have better grain quality and stronger host plant resistance. The fourth-generation MVs (MV4) were released after 1995 and included varieties developed for adverse production environments [5].

Despite the development and release of MVs, however, only a few have been widely adopted by farmers. The choice of rice variety to plant depends on many reasons. High yield is often thought to be one of the main reasons, although this is not always the case. Early studies have shown that other factors such as farm size, tenure status, education, and access to extension services and credit were also major determinants of varietal adoption and diffusion. However, the impact of many of these factors was no longer significant at the later stages of the diffusion process [7]. The perceived qualities of the variety, as well as its marketability (price), are also important considerations [7–8]. The latter depends on the grain quality preferences of consumers and other actors in the rice value chain such as millers, traders, wholesalers, and retailers.

Over the years, several studies have looked into the adoption and diffusion of MVs in the Philippines and other parts of Asia covering the first few decades of the Green Revolution [2, 9–10] as well as MV replacement in recent years [5,11–12]. Around 30–40% of the total rice area in the Philippines was planted to new MVs and the average replacement rate was around 8 to 11 years, with faster adoption rates during the dry season (DS) in irrigated areas [5]. This could be because the yield effects of MVs were more pronounced during the DS under favorable conditions as is observed in other Asian countries like Vietnam [13].

Farmers replace older varieties with new ones which they think will provide more net benefits or advantages [14]. In addition, their perceptions on the attributes of rice varieties were shown to be the major factors determining adoption and use intensities in Sierra Leone [8].

Although several studies have looked into the farm level adoption of MVs, only a few have studied in detail the varietal characteristics of adopted and officially released varieties and how these change with time. An exception is the study conducted by Juliano [15] that assessed the grain quality of MVs released in the Philippines from 1990–2009. Here, we extended this work to cover MVs released before 1990 and from 2010 to 2013, and included the agronomic characteristics, and reaction to pests and diseases of both released and adopted rice varieties. Because the varietal choice of farmers is not restricted to the grain quality attributes of varieties alone, an assessment to include other traits can provide better information on which traits and characteristics are valued by farmers.

This study aims to (1) characterize the MVs released in the Philippines from 1966 to 2013, (2) analyze temporal changes in MV adoption pattern and replacement, and (3) identify important traits and characteristics of the MVs that were widely adopted by farmers. We used the results of farm household surveys conducted in the wet and dry seasons in selected years from 1966–2012 in Central Luzon, a major rice growing region in the Philippines. The results of this study can contribute to the development of improved rice varieties that are better targeted to farmers’ preferences and, hence, would have a higher potential for adoption by farmers in Central Luzon and similar areas. Likewise, similar studies can be conducted in other major rice growing countries.

## Data and Methods

### The study area

The study area covers six provinces from two administrative regions in the Philippines: La Union and Pangasinan in Region I (Ilocos) and Nueva Ecija, Pampanga, Bulacan, and Tarlac in Region III (Central Luzon). For the purpose of this study, however, the six provinces will be collectively referred to as Central Luzon.

In 2013, the total harvested rice area in the six provinces was 0.9 million ha (double-cropped areas are counted twice), 82% of which was irrigated. During that year, the average rice yield was 4.7 t ha^−1^ per cropping season which was 20% higher than the national average [1]. Rice is planted twice each year: wet-season (WS) rice from June/July to September/October and dry-season (DS) rice from December/January to March/April (IRRI-SSD Central Luzon loop survey). The average farm size in 2011–12 was 1.3 ha which was smaller than the average farm size of 1.7 ha in 1998–99 (IRRI-SSD Central Luzon loop survey).

### Data sets

Rice varieties released between 1990 and 2013 and their associated characteristics (agronomic, grain quality, and reaction to pests and diseases) were obtained from records of the Philippine Rice Research Institute (PhilRice) and also from online sources [16]. For older varieties released starting in 1966, varietal characteristics were obtained from the database of the International Network for Genetic Evaluation of Rice (INGER, http://inger.irri.org/released-varieties) and from other publications [17–19].

Inbred varieties were classified into five groups based on the year of release [5]. Although the earlier MV classification was based on year of release and distinct characteristics, an additional class, MV5, was included to distinguish the MVs released after 2005 without taking into account the differences in characteristics with MV4. Like in Launio et al. [5], hybrid varieties released from 1994 onwards were placed under a separate group regardless of year of release.

**Fig 1:**
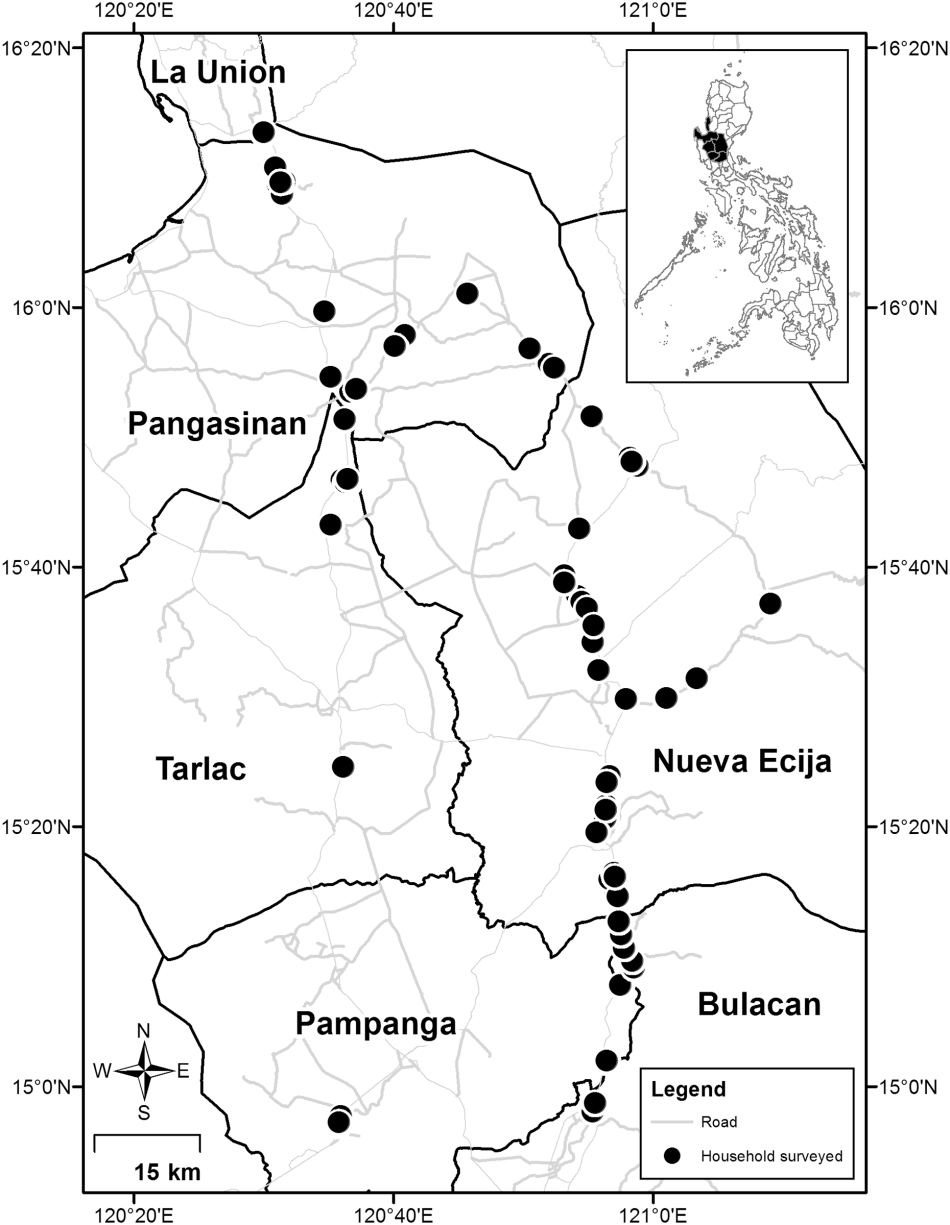
The study area showing location of farm households surveyed in 2011–12. The administrative map is based on the Global Administrative Areas database (GADM: http://www.gadm.org) and the road network from OpenStreetMap (http://download.geofabrik.de/asia/philippines.html).

We used data from seven farm household surveys conducted by IRRI's Social Sciences Division from 1966 to 2012 covering the following crop years: 1966–67, 1974–75, 1986–87, 1998–99, 2003–04, 2007–08, and 2011–12. Cropping years under the WS included 1966, 1974, 1986, 1999, 2003, 2008, and 2011, whereas those under the DS included 1967, 1975, 1987, 1998, 2004, 2007, and 2012. The farm households included in the sample are located along the “loop” of the main highway that passes through the six provinces (Fig 1). The respondents were informed that the survey was for research purposes and they gave verbal consent to be interviewed. Personal information (e.g., names and addresses) were collected to allow interviewers to return to the same household in another cropping season and survey year. Such information, however, were excluded from the survey files that were processed and analyzed.

### Rice varietal characteristics

We characterized rice varieties released from 1966 to 2013 and those planted by farmers using the following agronomic, grain quality, and pest and disease resistance traits:

#### Agronomic

(a)Yield (t/ha) is one of the most important considerations of farmers in choosing a variety to plant. This, together with farm gate price, determines the income from rice production. In this study, we considered average yields obtained from national field trials. (b) Maturity (days) refers to the number of days after sowing to harvest. Planting early maturing crops can help avoid critical adverse environmental conditions and allows the next crop to be planted earlier. (c) Plant height (cm); Taller plants are generally able to compete better with weeds but shorter plants are more resistant to lodging and have higher harvest index [20].

#### Grain quality

Several indicators are used to assess grain quality in rice [21], however, in this study we concentrated on milling recovery, proportion of head rice, grain length and shape, chalkiness, and amylose content which we deem are the most important grain quality traits. Moreover, information on these traits for the MVs released during the period covered in this study are readily available. Details about the specific methods used to measure the following grain quality traits are described by Juliano [15].

1. Milling recovery (%) is the proportion of the weight of milled rice to the weight of rough rice before milling. Varieties with high milling recovery give higher profits, hence, are more preferred by millers.
2. Head rice (%) is the proportion of milled grains that are at least three-fourths of the original grain length after complete milling. The difference between milled rice and head rice is the proportion of broken grains. Knowing the head rice value is important commercially as rice with less broken grains commands a higher price.
3. Grain length (mm) of 5.4 mm or less is classified as short, 5.5 to 6.5 mm is medium, 6.6 to 7.4 mm is long, and 7.5 mm and above is extra long. Grain length together with grain shape defines the appearance of the grain, which is important to consumers.
4. Grain shape is the ratio of the grain length to width. Rice with a grain shape of less than 2.0 is classified as bold, between 2.0 and 3.0 is medium, and more than 3.0 is slender. Grain shape contributes to the appearance of the grain but is less variable and less important to consumers than grain length [20].
5. Proportion of chalky grains (%); Although chalkiness disappears on cooking and has no effect on taste, excessive chalkiness downgrades the quality of the rice and consequently the price. A rice grain is chalky if at least 50% of it is opaque rather than translucent. Chalkiness also contributes to breakage during milling [15].
6. Amylose content (%) affects the cooking and eating quality of rice, as rice with a high amylose content (25–30%) tends to cook firm and dry, whereas rice with intermediate amylose content (20–25%) tends to be softer and stickier. Low-amylose (<20%) rice is generally quite soft and sticky. Glutinous rice, also referred to as waxy or sticky, has amylose content between 0 and 2% [22].

#### Reaction to pests and diseases

This refers to a variety’s reaction to pests/diseases such as rice blast, bacterial leaf blight (BLB), brown plant hopper (BPH), green leaf hopper (GLH), stemborer (deadheart/whitehead), and tungro (induced/modified) at the time of release. Information on this is indicated in the varietal release database as *susceptible (S)*, *moderately susceptible (MS)*, *intermediate (I)*, *moderately resistant (MR)*, *resistant(R)* or a combination of some or all (e.g., S to I, S to R).

### Analysis of survey data

We identified the most commonly planted rice varieties in Central Luzon during each survey season and year by ranking their percentage area and including only those varieties planted in the top 75% of area. We calculated the average values of the different rice varietal characteristics per varietal group weighted by area planted. In addition, the age of varieties was also calculated by subtracting the variety’s year of release from the survey year.

We compared the characteristics of released and adopted rice varieties across time. For the adopted varieties, we did pairwise comparisons of means by survey year using least significant difference (LSD). We analyzed the changes in the preferences of farmers for rice traits and characteristics and discussed the implications of these results on a more targeted development and dissemination of new rice varieties.

## Results and Discussion

### Rice varieties released in the Philippines

**Fig 2:**
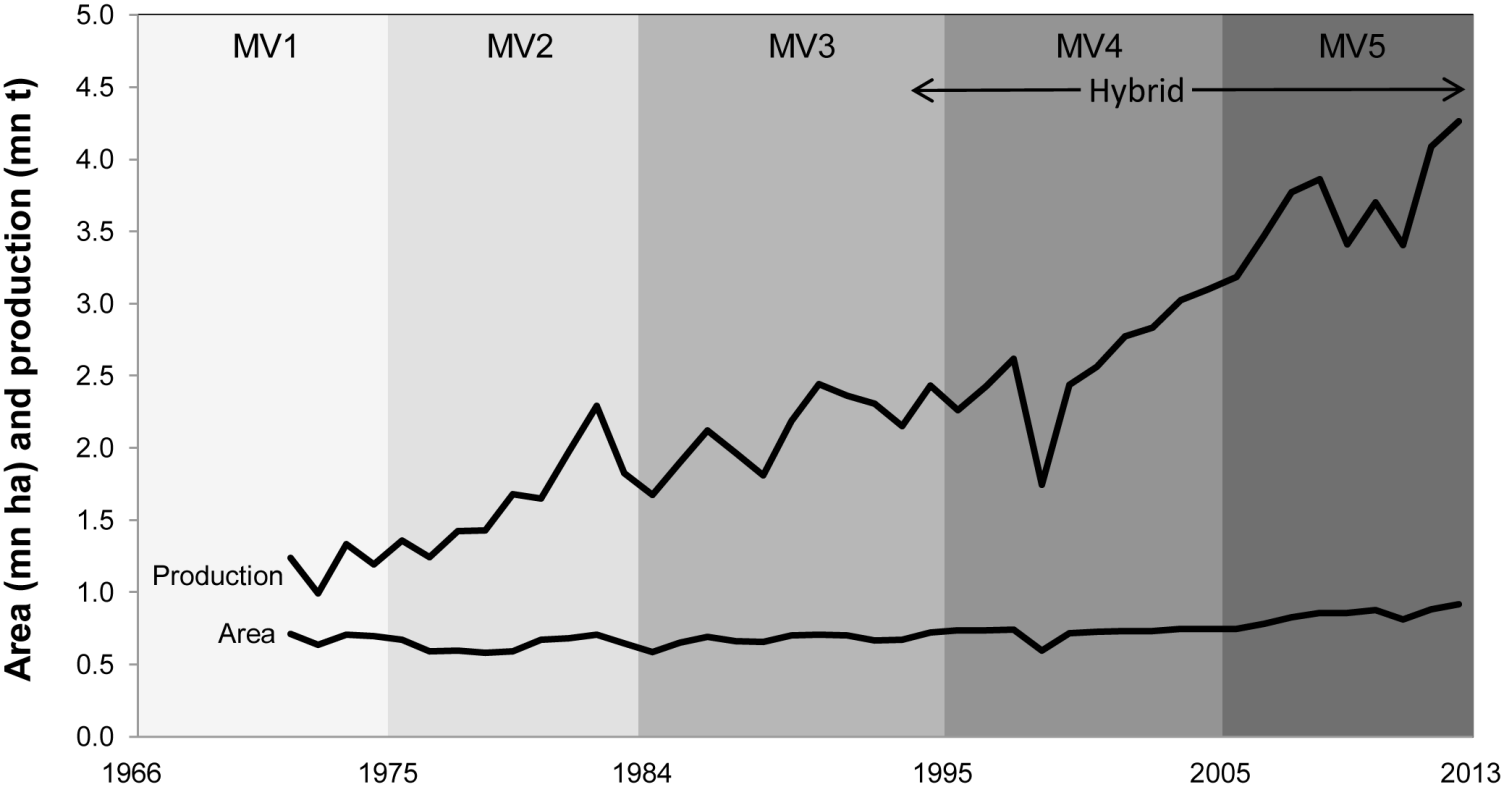
Total rice area and production in the six provinces covered by the Central Luzon Loop survey (Bulacan, La Union, Nueva Ecija, Pampanga, Pangasinan, and Tarlac) and periods of release of MVs by category. Source of area and production data: PSA-BAS (2015).

Fig 2 shows the total rice area (in mn ha) and production (in mn t) in the six provinces covered by the Central Luzon Loop survey and the period when the MV groups were released. Area planted to rice has stagnated since the mid 1960s but rice production continued to increase which could be due (in part) to the adoption of higher-yielding MVs. The rate of increase in production accelerated over the last decade which coincides with the release of MV4, MV5 and hybrid varieties.

The number of inbred MVs released in the Philippines increased from 18 varieties (MV1) to 65 (MV5) with 53 hybrids released from 1994 to 2013 (Table 1). MVs targeted for irrigated conditions dominated the released varieties across varietal groups/years. In later years, some varieties for adverse conditions such as saline-, drought-, and flood-prone areas were released.

**Table 1:** MVs released in the Philippines, 1966 to 2013^a^.

**Characteristic**	**MV1**	**MV2**	**MV3**	**MV4**	**MV5**	**Hybrid**
All	18	27	33	46	65	53
Glutinous	1	3	1	4	1	0
By target environment^b^						
Irrigated lowland	16	19	22	30	38	53
Rainfed lowland/Upland	2	8	9	12	14	0
Cool-elevated	0	0	2	4	0	0
Saline-prone	0	0	2	6	18	0
Drought/flood prone	0	0	0	0	2	0

^a^MV1: Varieties released from 1966 to 1975 including IR5 to IR34, and BPI varieties; MV2: Varieties released from 1976 to 1984 including IR36 to IR62; MV3: IR64 to IR74 and PSB RC released from 1985 to 1995; MV4: Varieties released from 1997 to 2005; MV5: Varieties released after 2005; Hybrid: Varieties released from 1994 to 2013.

^b^A variety can be classified into one or more target environments.

**Fig 3:**
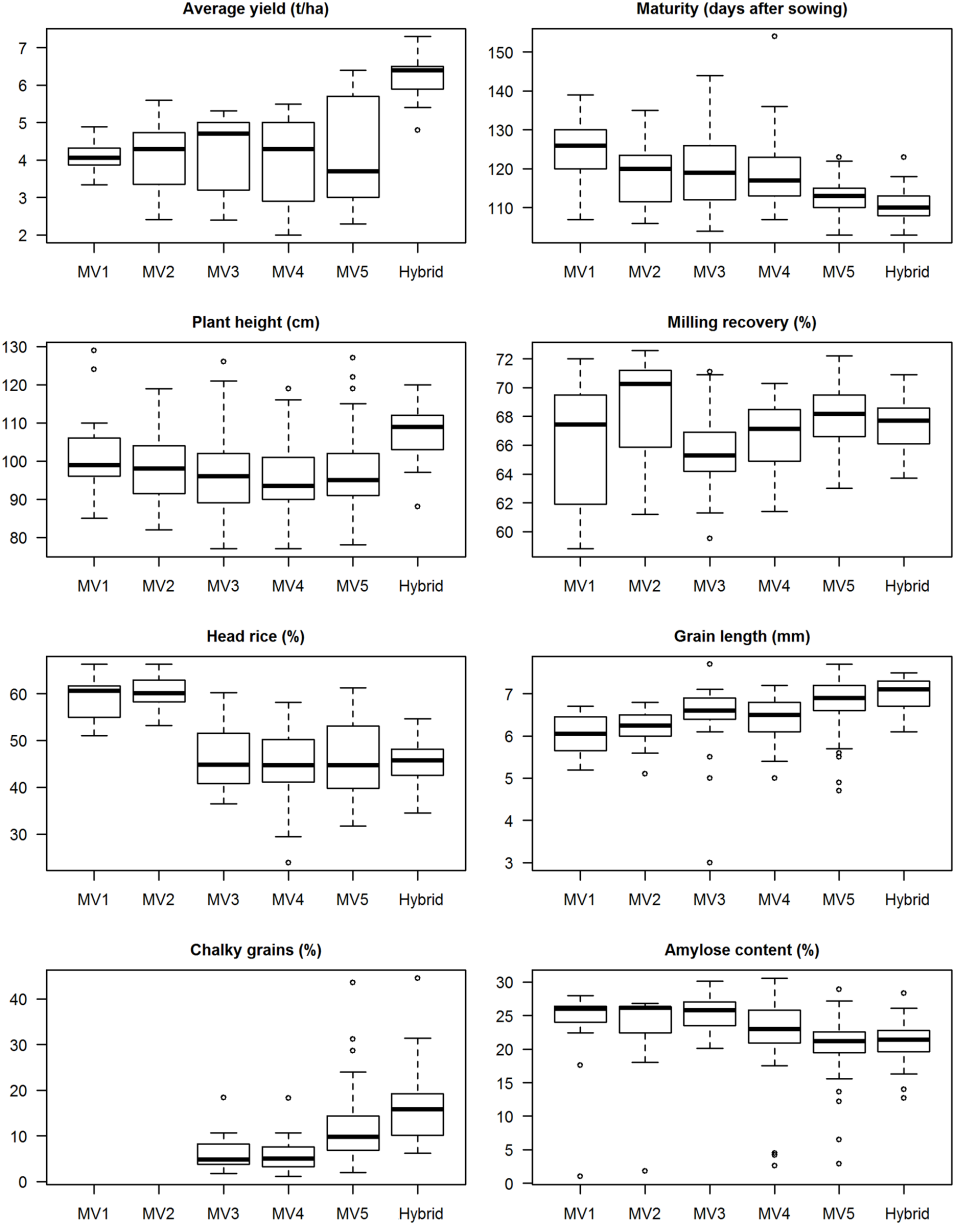
Agronomic and grain quality characteristics of MVs released from 1966 to 2013 by varietal group. See footnote in Table 1 for the varietal grouping description.

As expected, the average yield of hybrids was much higher than those of inbreds (Fig 3). The average yield increased from MV1 to MV3 but declined for MV4 and MV5. This is due to the release of several varieties which have lower yields (below 4 t/ha) but which could tolerate adverse conditions (cool elevated, saline-prone, and drought-prone). Median maturity also declined which means that, on the average, newer rice varieties have shorter growing periods. From 126 days for MV1, median maturity declined to 113 days for MV5 and 110 days for hybrids. The median plant height also declined slightly from 99 cm for MV1 to 95 cm for MV5. Hybrids were about 10 cm taller than MV1.

The milling recovery of MV2 varieties was higher than those of later generation MVs. Head rice was also significantly lower from MV3 to MV5 compared to earlier released varieties. Chalkiness was higher in MV5. Aside from genetic characteristics of the cultivar, chalkiness may also be due to high temperatures during grain development [20, 23] and infection by rice panicle blast and sheath blight [24]. Amylose content also declined with the release of new MVs that are softer when cooked. The median value for MV1 was 26% (high amylose), which dropped to 21.2% (intermediate) for MV5 and 21.4% for hybrids. These MVs reflect the breeding objective for grain quality in the Philippines and at IRRI, specifically for irrigated lowland ecosystems: long, slender, translucent grains that produce soft cooked rice [15], although this has changed at IRRI recently to reflect consumer demand in target areas [25–26].

**Fig 4:**
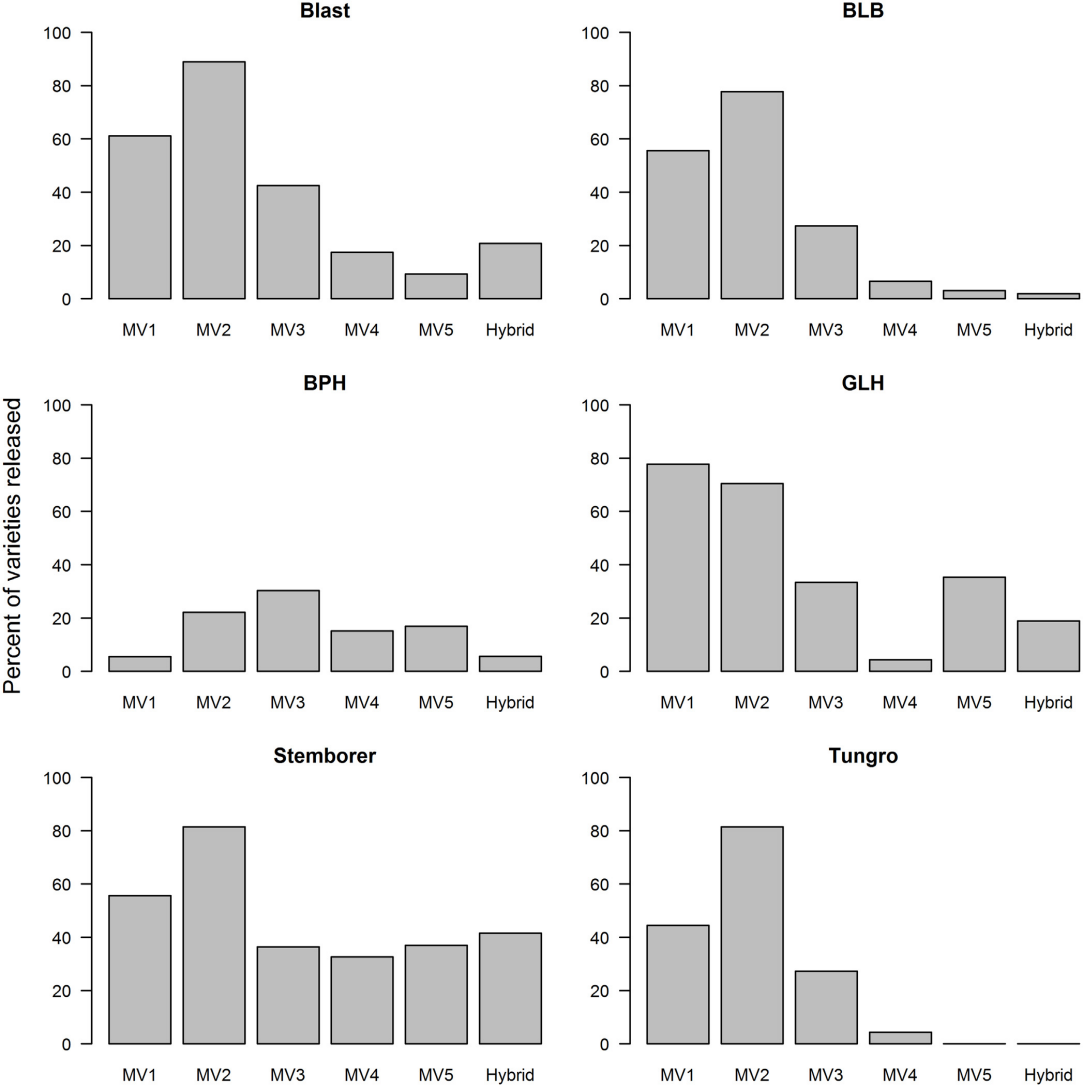
Proportion of MVs released from 1966 to 2013 that have resistance to pests/diseases by varietal group. See footnote in Table 1 for the varietal grouping description.

A higher proportion of early MVs (MV1 and MV2) has a broader spectrum resistance to pests and diseases compared to more recently released varieties (Fig 4). MV2 has the highest proportion of varieties with multiple pest and disease resistance. In contrast, more than 80% of MV5 varieties have no resistance to blast, BLB, BPH, and tungro, which are among the common pests and diseases in rice in the Philippines. The decline in disease resistance in recently released varieties is likely due to the successful breeding and release of disease-resistant rice varieties that started in the 1960s and 1970s [27]. With the decrease in disease outbreaks, the emphasis shifted to the breeding of varieties that are tolerant to adverse conditions. However, due to climate change, shifts in plant pests and diseases are anticipated with some becoming more damaging [28]. These shifts should be taken into account in the development of new varieties.

### Rice varieties cultivated by farmers in Central Luzon

#### Adoption pattern and replacement

From 1966 to 2012, the number of unique rice varieties grown by farmers in the study area ranged from 27 to 47 (Table 2). Varietal richness is higher during the WS than in the DS, and in earlier years the total DS area is much lower. However, in 2011–12 the number of varieties planted during the WS was double those planted during the DS even though the area difference was not as much. Less than 10 varieties occupy the upper 75^th^ percentile of rice area during each survey year. There were early adopters of newly-released varieties but there were also farmers who still planted varieties that were released more than ten years prior to the survey. There were varieties already planted in farmers’ fields on the same year or even prior to official release. Launio et al. [5] also observed this and further explained that this is possible because some varieties were already submitted for seed multiplication but the official approval was delayed. Also, some farmers’ selection varieties (e.g., NSIC Rc 122) were already planted in farmers’ fields long before they were officially released.

**Table 2:** Rice varieties planted by farmers in Central Luzon, Philippines, 1966–2012.

**Characteristic**	**1966–67**	**1974–75**	**1986–87**	**1998–99**	**2003–04**	**2007–08**	**2011–12**
**Number of unique varieties planted**							
All year	32	27	47	38	34	46	45
Wet season	31	22	37	27	27	35	37
Dry season	7	11	21	25	20	28	18
Top 75%	10	7	7	8	7	9	9
**Most popular variety**	Tjeremas	IR 20	IR 64	IR 64	PSB Rc82	NSIC Rc128	NSIC Rc216
Intensity (% area)	20	36	49	30	34	20	19
Incidence (% farmers)	13	30	34	28	28	18	20
**Year of release of MVs adopted**							
**Most popular**	TV	1969	1985	1985	2000	2004	2009
**Newest**	All TVs	1978	1987	1998	2003	2007	2010
**Oldest**	All TVs	1969	1969	1985	1985	1994	2000
**Weighted average age of MVs planted**	_	4.3 (WS)	4.6 (WS)	8.8 (WS)	5.2 (WS)	6.4 (WS)	2.8 (WS)
	_	2.6 (DS)	3.4 (DS)	9.0 (DS)	7.5 (DS)	3.8 (DS)	4.7 (DS)
Number of respondents	95	59	120	85	116	107	95
Area surveyed (ha)	165 (WS)	149 (WS)	206 (WS)	127 (WS)	202 (WS)	118 (WS)	145 (WS)
	25 (DS)	29 (DS)	111 (DS)	71 (DS)	129 (DS)	85 (DS)	119 (DS)

The weighted average age of MVs grown by farmers was 4.3 years in the WS and 2.6 in the DS in the mid 1970s. This more than doubled in the WS and tripled in the DS in the 1998–99 surveys as a result of a large area planted to IR64 which was released in 1985. The varietal age of the adopted MVs decreased in later years. Until 2007–08, the varietal age in the DS was generally lower than in the WS which is consistent with the findings of Launio et al. [5]. This trend, however, reversed in 2011–12 when the adoption of newer varieties became faster in the WS. This was due to the large areas planted to varieties NSIC Rc 216 and NSIC Rc 222 which were both released in 2009 –only 2 years prior to the WS survey.

Hybrids are expected to be more popular during the DS when the risks of pests and diseases and harsh weather conditions like typhoons are low [29]. However, despite the 53 hybrid varieties released since 1994, only NSIC Rc 132H (Mestiso 6), released by a private company in 2004, was widely adopted during the survey. This is the only hybrid variety planted in large areas (25% of the survey area) by the farmer respondents in the 2012 DS and no hybrid variety was planted in the 2011 WS in our survey area. Based on the farm survey, out of the 15 farmers who planted this hybrid variety in the 2012 DS, 12 obtained the seeds from the Department of Agriculture. This suggests that a seed distribution program was in place at that time which facilitated the adoption of this particular variety.

Prior to the 2012 DS, adoption of hybrid rice was low despite seed subsidies and heavy promotions by the government. Based on analysis of data at the municipal level in various provinces from 2002 to 2004, David [29] concluded that majority of farmers have not been convinced about the economic advantage of growing hybrid rice over the best yielding inbreds. Labor use particularly for seedbed preparation and crop establishment was found to be higher for hybrids than for inbreds. Moreover, early hybrid varieties were deemed inferior in terms of grain quality compared with best inbreds [30]. The low adoption of hybrids despite its proven yield advantage implies that high yield is not always the main consideration of farmers in choosing a variety to grow.

**Fig 5:**
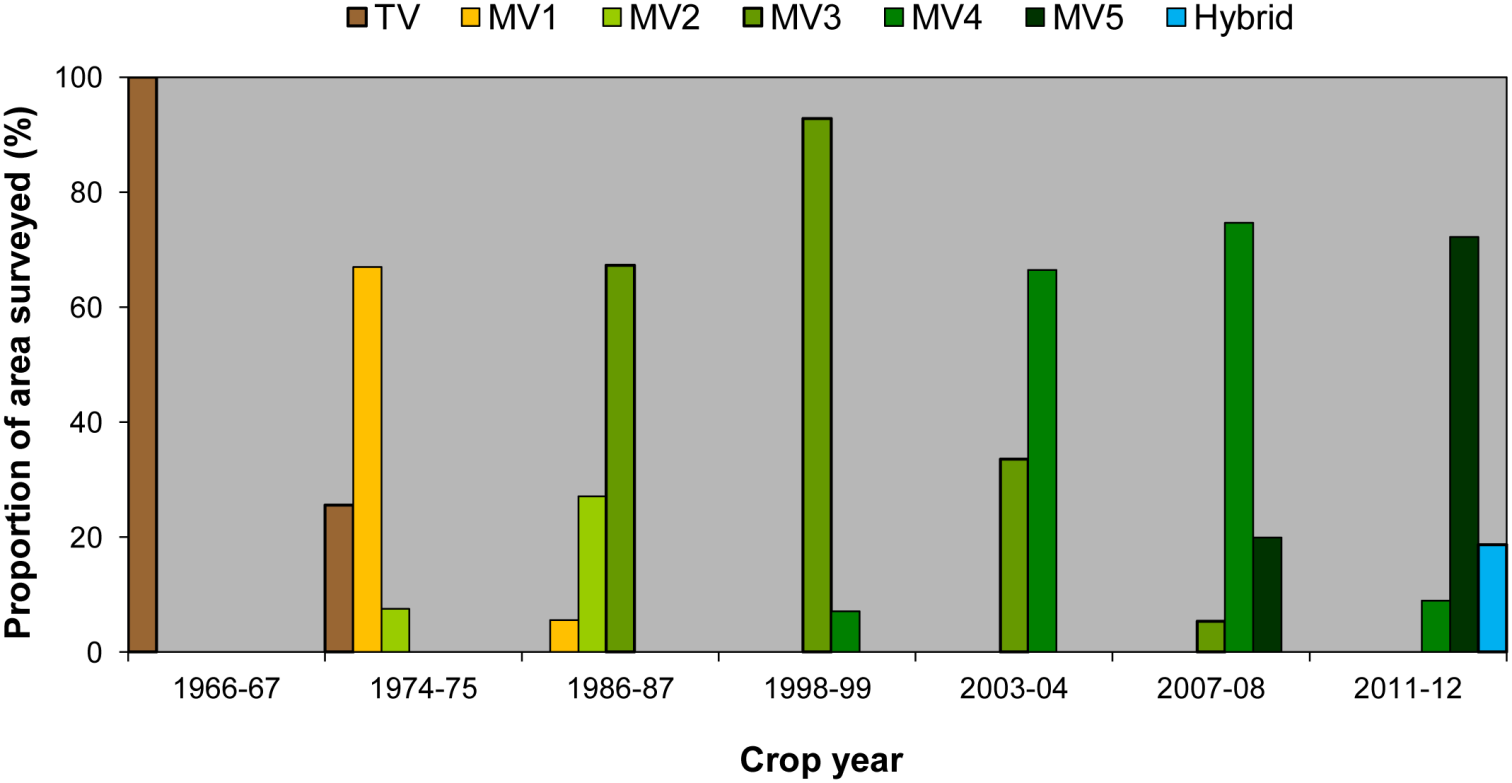
Adoption of rice by varietal group and crop year when the survey was conducted. See footnote of Table 1 for the varietal grouping description.

As expected, no rice area was planted to MVs in the study site during the 1966–67 survey year (Fig 5). After a decade, MVs were planted on 74% of the total rice area, increasing to 100% by the mid 1980s. By 2007–08, 20% of the rice area was already planted to MVs that were released after 2005 and increased to 75% by 2011–12. The varietal group MV3 was the most widely grown for over 10 years because of IR64. Based on the survey years, adoption of MV3 varieties peaked in 1998–99 (93%), MV4 in 2007–08 (75%), and MV5 in 2011–12 (75%).

Among the top varieties planted by farmers in Central Luzon, IR64 had the highest adoption intensity and incidence in the 1986–87 and 1998–99 survey years (Table 2). In the mid 1980s, half of the survey area was already planted to IR64. Twelve years later, IR64 was still planted to 30% of the survey area and five years hence was still grown on 5%, making it the most widely adopted MV in Central Luzon in terms of extent of area covered and number of years grown. Moreover, the variety PSB Rc82, the most popular variety in 2003–04 which was grown on 34% of the survey area, is also a progeny of IR64.

Based on a survey of farmers in Nueva Ecija during the 1987 WS, their top reasons for planting IR64 were its good yield (42% of respondents), good eating quality (24%), and disease and pest resistance (17%) [31]. On the average, IR64 yields 3.9 t ha^-1^ in the WS and 6.3 t ha^-1^ in the DS, matures at 117 days, and has a plant height of 103 cm [19]. It has intermediate amylose content (23.2%) and long and slender grains. In addition, IR64 was moderately resistant to blast and stemborer, and resistant to BLB, BPH, GLH, and rice tungro disease at the time of release [19].

In this study, we assumed that the availability and accessibility of seeds do not constrain the varietal choice of farmers in Central Luzon. This assumption is justified considering the proximity of the study area to PhilRice, a major rice breeding institution in the Philippines which produces foundation seeds of newly-released varieties [5]. To facilitate the distribution of seeds of MVs especially in other areas, the current seed system in the Philippines needs to be strengthened. The low farmgate price of seeds coupled with the high cost of production and distribution do not provide enough incentives to increase seed production [32]. These need to be addressed to make new MVs more accessible to farmers.

#### Characteristics of adopted MVs

**Fig 6:**
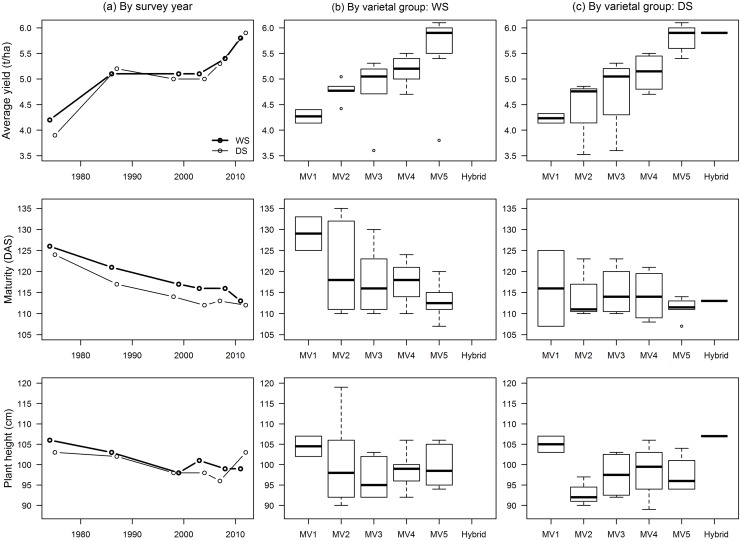
Agronomic characteristics by survey year (a), and varietal group (b,c) of MVs adopted by farmers in Central Luzon, 1974–2012. See footnote in Table 1 for the varietal grouping description.

The weighted average yield of MVs adopted by farmers increased from 4.1 t ha^-1^ in 1974–75 to 5.1 t ha^-1^ in 1986–87 and did not change in the next two survey years. It slightly increased to 5.3 t ha^-1^ in 2007–08 and then to 5.8 t ha^-1^ in 2011–12 (Table 3). Farmers adopted only MVs with average yields of at least 3.5 t ha^-1^ across the survey years, and MVs that have high yields were planted to more areas (Fig 6). Adopted MV5 had yields of at least 5.4 t ha^-1^ except for one variety, NSIC Rc 218 SR (Mabango 3), an aromatic rice which has an average yield of 3.8 t ha^-1^ and was planted to 3% of the rice area in the 2011 WS. In general, older MVs were replaced with newer ones that have higher yields. Although there were differences in varieties adopted by season, the weighted average yields of adopted varieties were not significantly different between seasons.

**Table 3:** Agronomic and grain quality characteristics of rice varieties planted by farmers in Central Luzon, by survey year (weighted by area), 1974–2012^a^.

Characteristics	1974–75	1986–87	1998–99	2003–04	2007–08	2011–12
*Agronomic*						
Average yield (t/ha)	4.1 d	5.1c	5.1 c	5.1 c	5.3b	5.8a
Maturity (DAS)	126a	119b	116c	115 d	115 d	113 e
Plant height (cm)	106a	103b	98e	100d	98e	101c
*Grain quality*						
Milling recovery (%)	67.6^bc^	69.8a	67.7bc	67.9b	67.3c	67.9b
Head rice (%)	60.5^**a**^	60.0a	52.4b	46.1c	44.7d	45.7c
Grain length (cm)	5.49^f^	6.56e	6.72d	6.78c	6.84b	7.15a
Grain shape (ratio)	2.61^d^	3.19c	3.25ab	3.23b	3.26a	3.25ab
Chalky grains (%)	-	-	3.4d	4.9c	7.9b	13.0a
Amylose content (%)	25.5a	24.1b	22.5c	22.0d	20.3e	20.5e

^a^Values in a row with the same letter are not significantly different at 5% level of significance.

The maturity of adopted MVs decreased significantly across the survey years (Table 3; Fig 6), with maturities across varietal groups ranging from 107 to 135 days. MVs adopted during the DS matured faster than those grown in the WS except during the last survey year when the gap closed. For the last four DS survey years, weighted average maturities ranged from 112 to 114 days. The average maturity of adopted MV5 varieties ranged from 107 to 120 days.

The plant height of adopted MVs also decreased with the weighted average at 100 cm in the last three survey years (Table 3), ranging from 89 to 119 cm (Fig 6). The range is shorter for adopted MV5 varieties: 94 to 106 cm.

**Fig 7:**
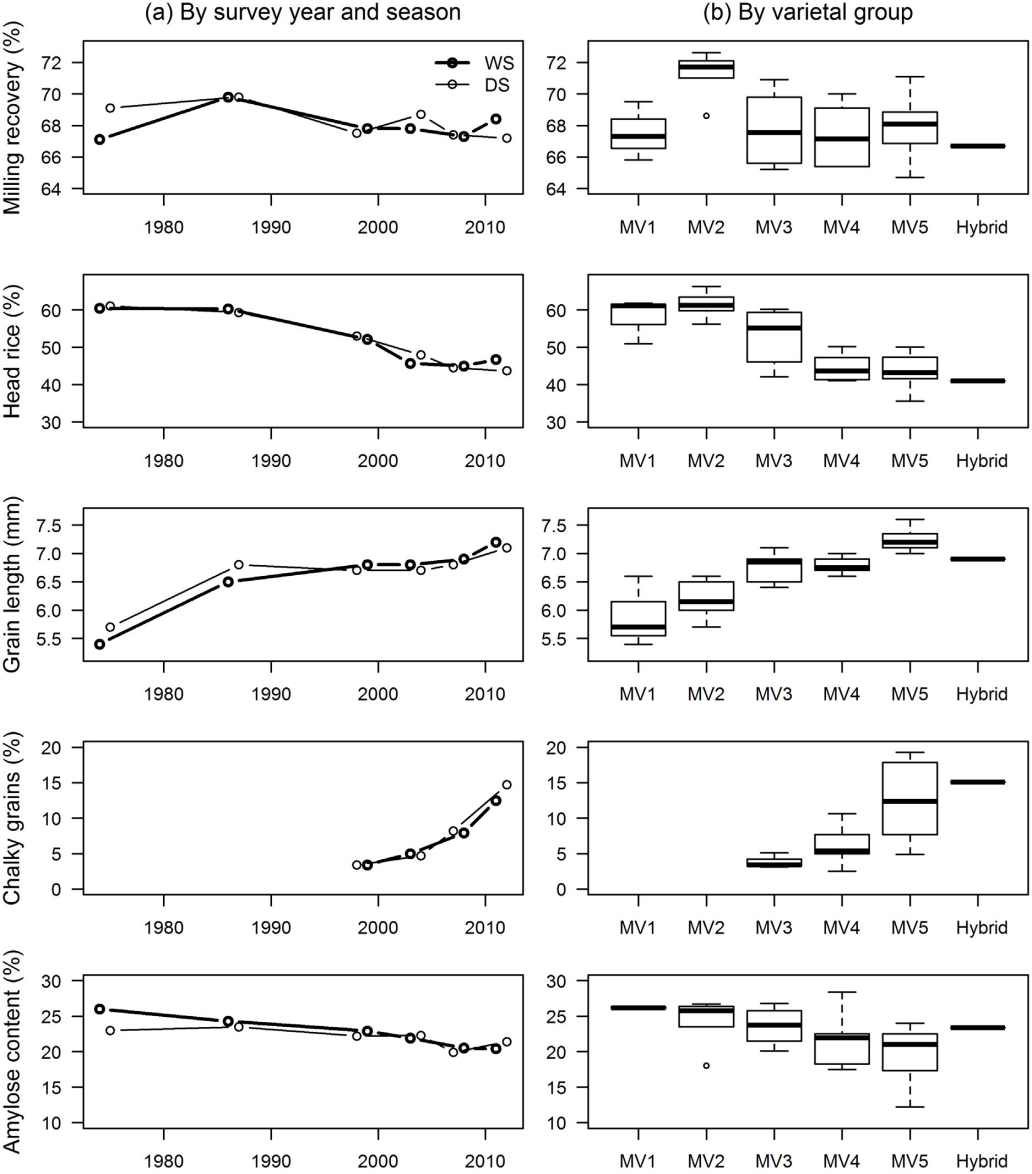
Grain quality by survey year and season (a), and by varietal group (b) of MVs adopted by farmers in Central Luzon, 1974–2012. See footnote in Table 2 for the varietal grouping description.

The weighted average milling recovery of MVs planted by farmers did not change much across the survey years (Table 3), ranging from 65% to 73%, with the milling recovery of the adopted MV2 varieties much higher (Fig 7). Head rice, however, decreased substantially from 61% in the mid 1970s to only 46% in the most recent survey year. The head rice of adopted MVs ranged from 36% to 66%, but for MV5 the highest head rice was only 50%. Mackill et al. [20] suggested that more emphasis be given to the improvement of head rice than total rice yield as it is more important commercially, it varies more, and it is easier to improve.

Grains of MVs planted by farmers used to be of medium length and intermediate shape in the mid 1970s (Table 3). These changed to long and slender from the mid 1980s onwards. Grain length of cultivated MVs significantly increased in each survey year but shape did not change much since the mid 1980s. The proportion of chalky grains in the adopted MVs nearly quadrupled from only 3.4% in 1998–99 to 13% after 14 years. The amylose content of the adopted MVs across the survey years ranged from 12.2% (low) to 28.4% (high) (Fig 7). The weighted average of the amylose content of MVs planted by farmers in the mid 1970s was 25.5% (high) which decreased to 20.5 (intermediate) in 2011–12 (Table 3). Old MVs were replaced with newer ones that produce softer cooked rice for consumption.

Grain quality is determined by the kind of variety planted but is also affected by the production environment and processing [33]. Head rice, for instance, is determined by the variety’s genetic makeup but is also affected by drying and milling techniques. Similarly, location and season affect amylose content and other grain quality traits [15]. Changes in measurements across years may have been affected by factors other than the intrinsic characteristics of the variety. Although these are important considerations, we limited our analyses to the characteristics of varieties as measured during national cooperative trials.

**Fig 8:**
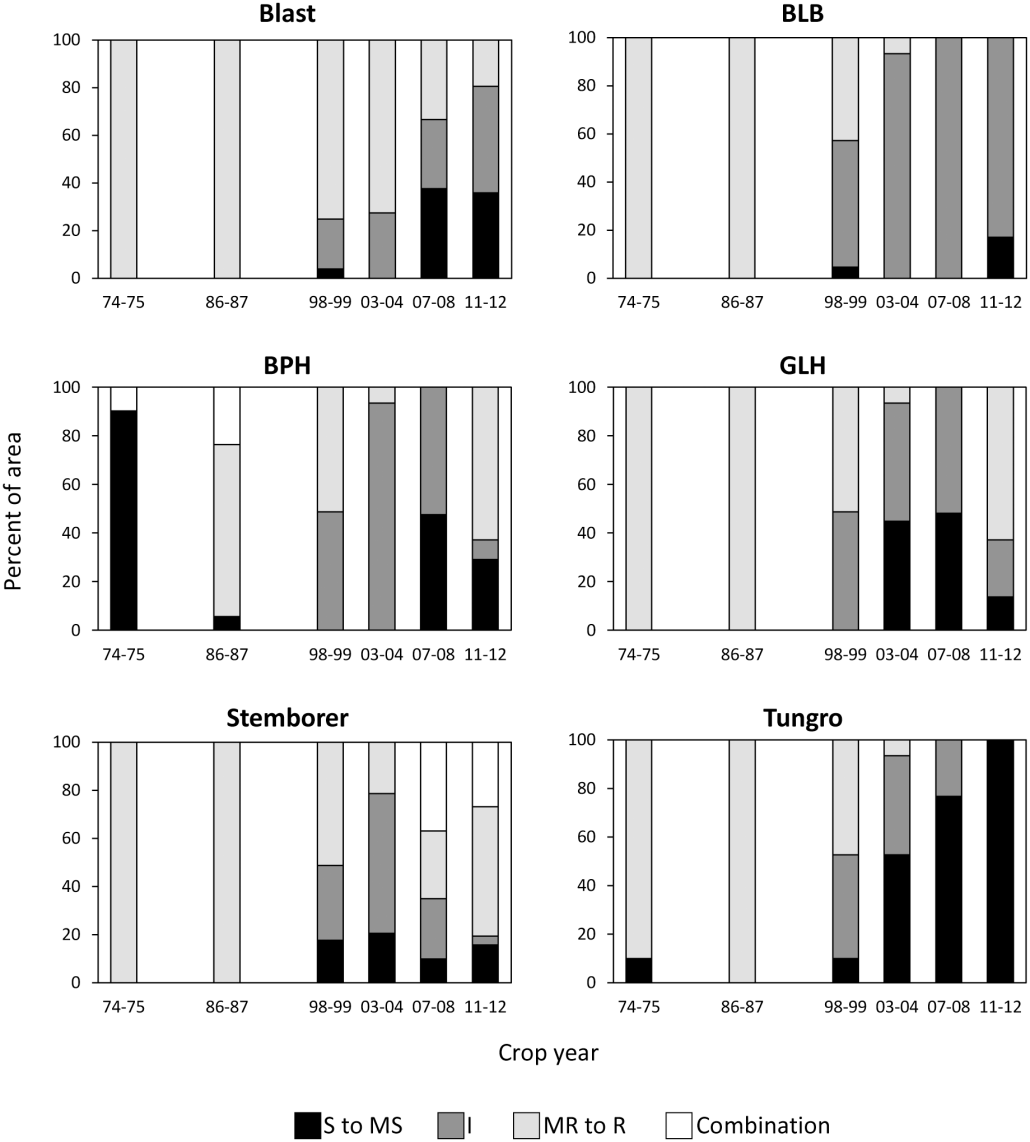
Pest and disease reactions of rice varieties planted by farmers in Central Luzon, by survey year, 1974–2012. S to MS: susceptible to moderately susceptible; I: Intermediate; MR to R: moderately resistant to resistant.

Based on pest and disease resistance ratings at the time of release of the rice varieties, the proportion of the rice area planted to varieties that are moderately susceptible to susceptible to blast, BLB, and tungro increased (Fig 8). Similarly, the proportion of rice areas planted to MVs that are moderately susceptible to susceptible to GLH increased from none in the mid 1980s to 48% in 2007–08 but declined to 14% in 2011–12. In the case of BPH, the area planted to susceptible/moderately susceptible varieties reduced from 90% in the mid 1970s to only 6% in the mid 1980s. This increased again to 47% in the last decade.

In the last 3 survey years, all or nearly all areas were planted to MVs that are either susceptible or moderately susceptible to tungro compared with only 10% in the 1970s. In the 2011–12 crop year, all varieties adopted by farmers were susceptible to tungro. From a survey of rice farmers in Central Luzon in 1990, 67% mentioned tungro as a problem and 26% mentioned BPH [34]. The same findings were reported by another study in which tungro was also identified by many farmers in Central Luzon as a problem in their rice fields [35], although a more recent study indicated low levels of incidence of tungro in this region [36]. These were also the same observations made during in-field surveys conducted by the Philippine Bureau of Plant Industries from 1993 to 2013 (W. Cuaterno, personal communication). However, less than 4% of MVs released after 1995 (MV4, MV5, and Hybrid) are resistant to tungro (Fig 4), thus, giving farmers very limited options.

A variety’s resistance to pests and diseases breaks down after several years. The genes responsible for such resistance are often rendered ineffective a few seasons after their release due to the pest or pathogen overcoming the resistance mechanisms. This was not accounted for in this analysis because of the difficulties in determining when resistance genes in plants are overcome because these events occur at different rates depending on each pest or pathogen and host interaction.

Prioritizing the resistance traits to be considered in breeding for a particular target area is important along with the identification of new sources of resistance. As more resistance traits are included, the difficulty to recombine all desired genes into a single line increases [20].

## Summary and Conclusions

High yield, good grain quality, and resistance to pests and diseases are some of the most important characteristics that farmers consider when choosing a rice variety [5]. The analysis of five decades of farm household survey data in Central Luzon provides insights into the varietal characteristics preferred by farmers and how these change with time. This can serve as a guide in identifying the combinations of traits that will have a high probability of being widely adopted by farmers in Central Luzon and similar areas.

Farmers in Central Luzon prefer varieties that give high yield, mature faster (shorter growing season), and have long and slender grains, high milling recovery, and intermediate amylose content. The level of amylose content of adopted varieties has been declining, consistent with the recommendation of Unnevehr et al. [37]. This study suggests that there is value in further developing new varieties with intermediate amylose content or softer rice. New varieties to be developed and disseminated in Central Luzon should have these characteristics for them to have a high potential for adoption. The trends in characteristics over time of adopted varieties in terms of maturity, grain length and amylose content, and those of released varieties follow a similar pattern. This suggests that rice breeders in the Philippines had been successful in developing varieties that respond to farmers’ preferences for these traits.

In addition, new varieties should have higher head rice recovery, less chalky grains, and better resistance to pests and diseases. Recently developed varieties compared poorly in these traits. Low head rice and chalkiness negatively affect price and, hence, the income of farmers. Moreover, to reduce the risks of severe outbreaks, a broad spectrum resistance should be incorporated into new varieties similar to that of the second-generation MVs.

Over 200 MVs have already been developed and released in the Philippines, yet each crop year, only less than 10 varieties are planted in 75% of the total area planted to rice in the study area. The development of varieties entails a lot of resources and takes a considerable amount of time. Understanding the characteristics of widely adopted varieties can contribute to the development of better targeted varieties that would have a high probability for adoption. This would allow better optimization of the use of limited funds for breeding programs. Insights from this study can also benefit other similar rice producing regions in the country. Further, we recommend a similar analysis be done to look into the characteristics of varieties adopted by farmers in other major rice growing regions to aid the development of MVs that are better responsive to the needs and preferences of farmers.

Although varietal adoption is dependent on many factors, here we have attempted to look at characteristics of MVs in terms of agronomic, grain quality, and stress resistance particularly to pests and diseases. Surveys on consumers, farmers and other rice value chain actors are currently being conducted to gain a deeper understanding on their preferences for rice traits and how these vary by location, urbanity, and socio-economic class. Moreover, rice environments are being characterized to identify which areas are prone to stresses as the agroclimatic environment has been shown to be the most significant determinant of geographical differences in adoption rates [7]. Results from this further analysis can contribute to the development of rice product profiles per region and a better targeted breeding program.
